# Supplementary material for: EpicTope: predicting and validating non-disruptive epitope tagging sites
Source: Development. 2026 Mar 12;153(5):dev204502. doi: 10.1242/dev.204502 (PMC13006528; doi:10.1242/dev.204502)
Supplement: Supplementary information [file develop-153-204502-s1.pdf]

Supplementary Materials:

The raw data scores for Smad5 and Hdac1 proteins are provided on Figshare https://doi.org/10.6084/m9.figshare.28836158

The EpicTope software is available on GitHub, https://github.com/FriedbergLab/Epictope

The EpicTope source code can be found at Zenodo, https://doi.org/10.5281/zenodo.17957808

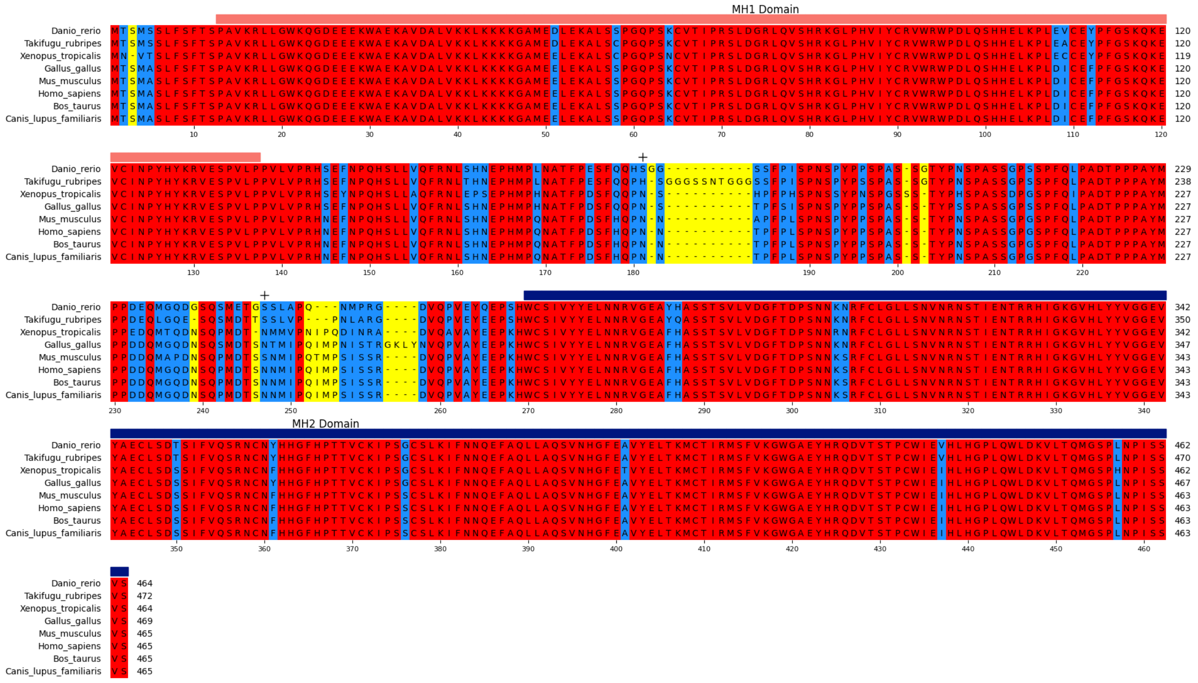

Fig. S1. Multiple Sequence Alignment for *Danio rerio* Smad5. Amino acids identical between all species are highlighted in red and differences are highlighted blue. Amino acid length variation is shown in yellow. Location of internally inserted *Danio rerio* Smad5 tags at S181 and 247 are labeled with a black "+". MH1 and MH2 domains are indicated by horizontal red and dark blue bars, respectively. Total length of each Smad5 protein is shown at the end of the alignment, and position indices are labeled in reference to *Danio rerio*.

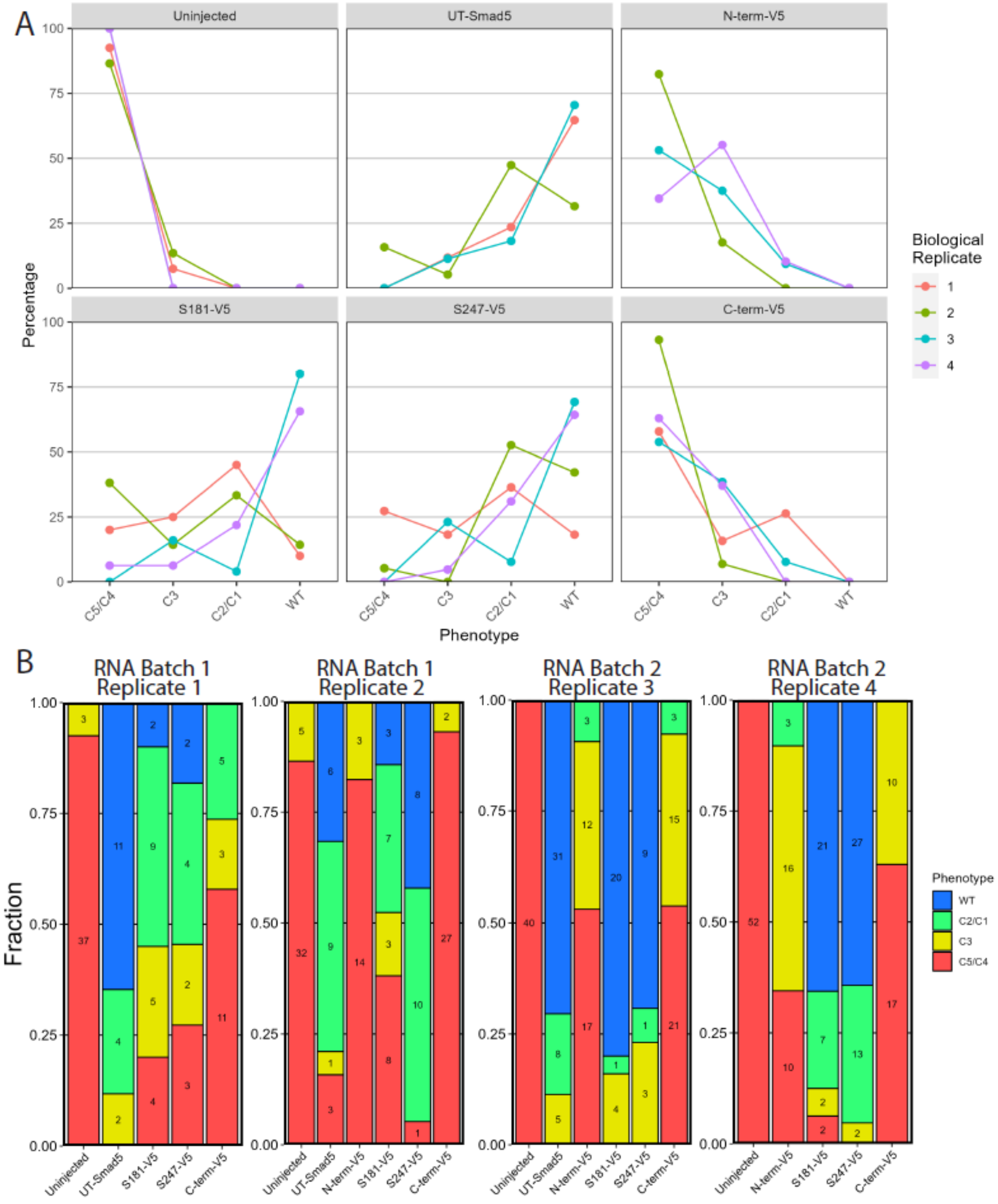

Fig. S2. Biological Replicates of V5-Tagged Smad5 Rescue. Quantification of *smad5*^{dtc24} mutant embryos injected with 150 pg of untagged (UT) or V5-tagged *smad5* RNA. The dorsalized classes C1-C5 are the scoring scale in Mullins et al, 1996. A) Points on the lines are the percent embryos with the phenotype specified on the x-axis. Different colored lines are different biological replicates. B) Bar graphs of data in A with the number of embryos noted in each bar.

Development • Supplementary information

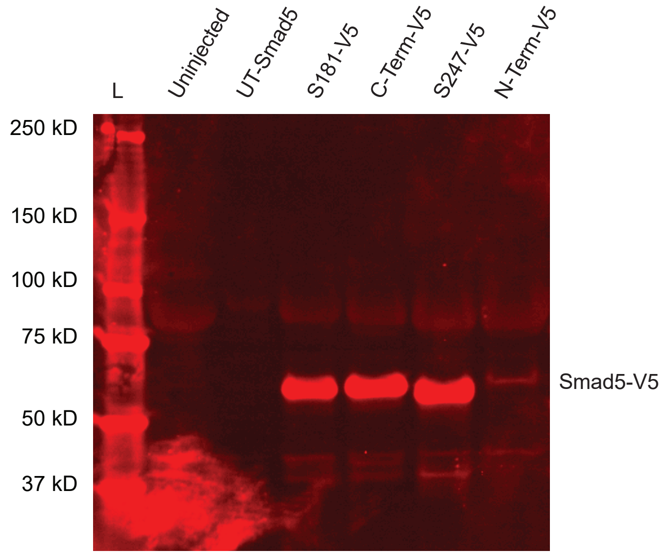

Fig. S3. Digitally adjusted red (V5) channel from the western blot in Fig. 3 to show the N-terminal V5-tagged Smad5 band. The upper limit of the brightness display range was lowered to 23 from 255. Embryos were injected with V5-tagged or untagged (UT) mRNAs. Seven 6 hpf (shield stage) embryos were used in each lane. L is the molecular weight ladder.

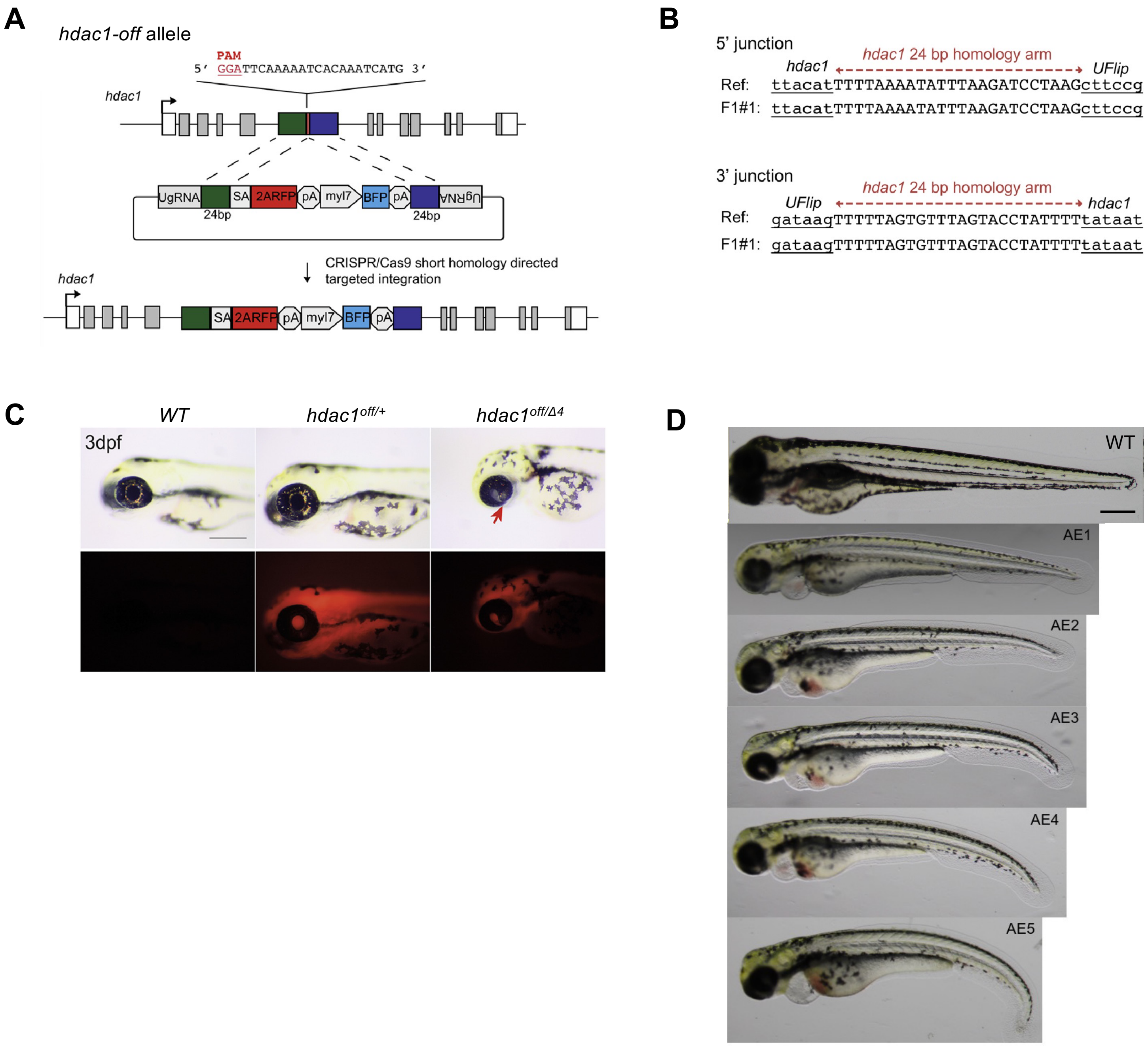

Fig. S4. Zebrafish loss of function *hdac1*^{is65off} knock-in allele A) Diagram of *hdac1*^{is65off} loss of function KI allele. B) 5' and 3' genomic-UFlip cassette integration junctions were PCR amplified from F1 transgenic zebrafish fin clip genomic DNA. The PCR products were sequenced and aligned to the reference sequence expected for a precise integration at the genomic target site. C) *hdac1*^{is65off} KI allele showing mRFP expression. Gross phenotype of 3 dpf transheterozygous *hdac1*^{is70del4}/*hdac1*^{is65off} larva showing microcephaly and retinal coloboma (arrow). D) Embryos displaying all scoring categories show the range of axial extension defect phenotypes in transheterozygous *hdac1*^{is70del4}/*hdac1*^{is65off} 4 dpf zebrafish embryos. The WT, A2, A3 and A5 images are from Fig. 7B. Scale bars C,D 200 μM.

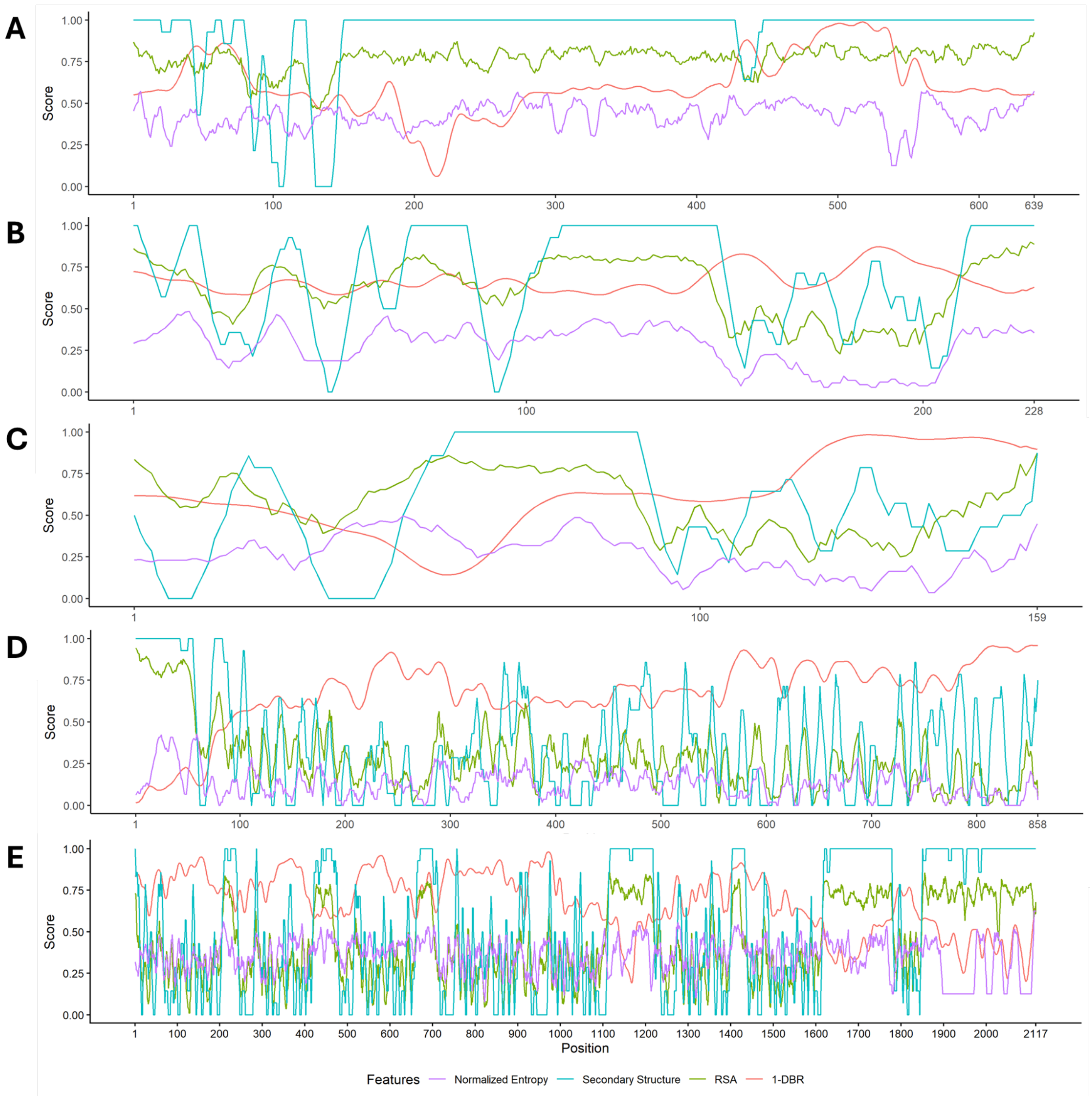

Fig. S5. EpicTope feature scores for additional proteins. Shown are Shannon entropy, secondary structure, relative solvent accessibility (RSA) and disordered binding region (DBR) feature scores across five proteins (A) Buc (H0WFA5), (B) Nanos1 (E7FDB3), (C) Nanos3 (Q90WW1), (D) Piwil1 (Q8UVX0), and (E) Tdrd6 (F1R237). Features are normalized on a 0-1 scale, and plotted by averaging along a sliding window of 7 amino acids, except at N- and C- termini where the 4-6 terminal amino acids were used.

Development • Supplementary information

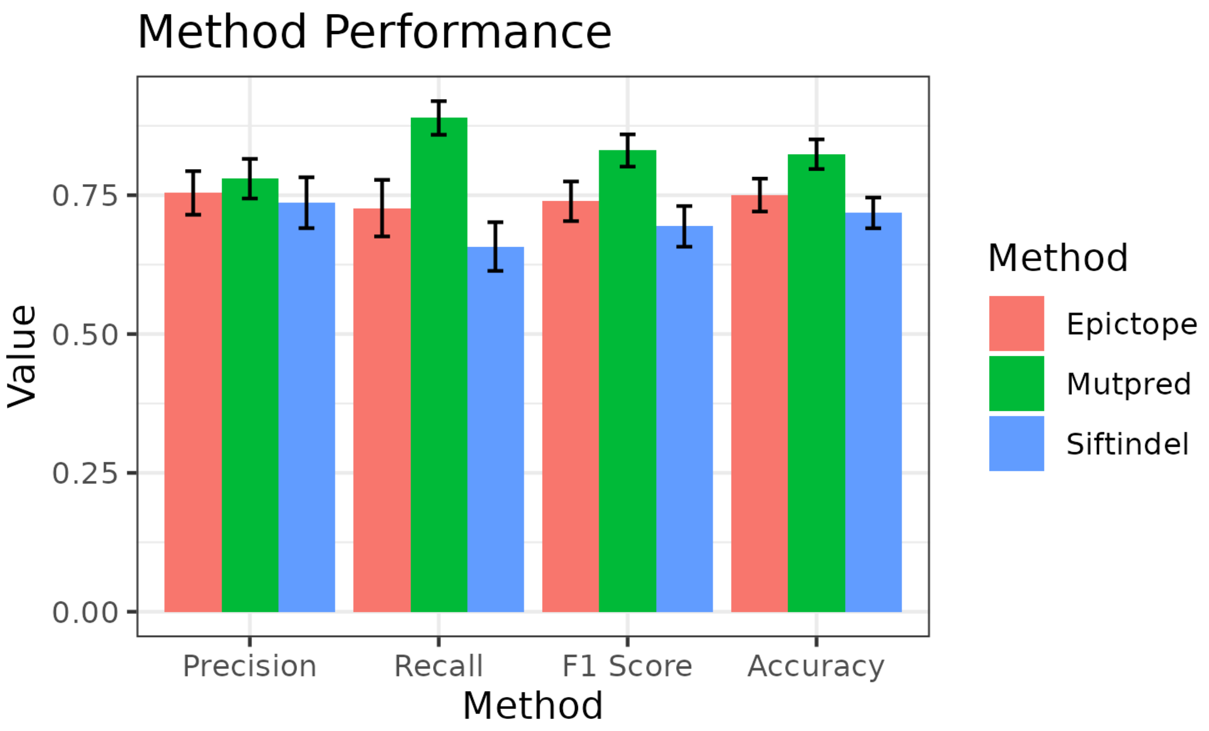

Fig. S6. F1 Score Comparison. Shown are the precision, recall, F1 and accuracy scores for SIFT-indel, EpicTope, and Mutpred. Error bars indicate standard deviation over 100 replicates of the testing set. SIFT-indel achieved an F1 score of 0.694 (sd = 0.036), EpicTope achieved a score of 0.739 (sd = 0.035), and MutPred2 a score of 0.831 (sd = 0.029).

Development • Supplementary information

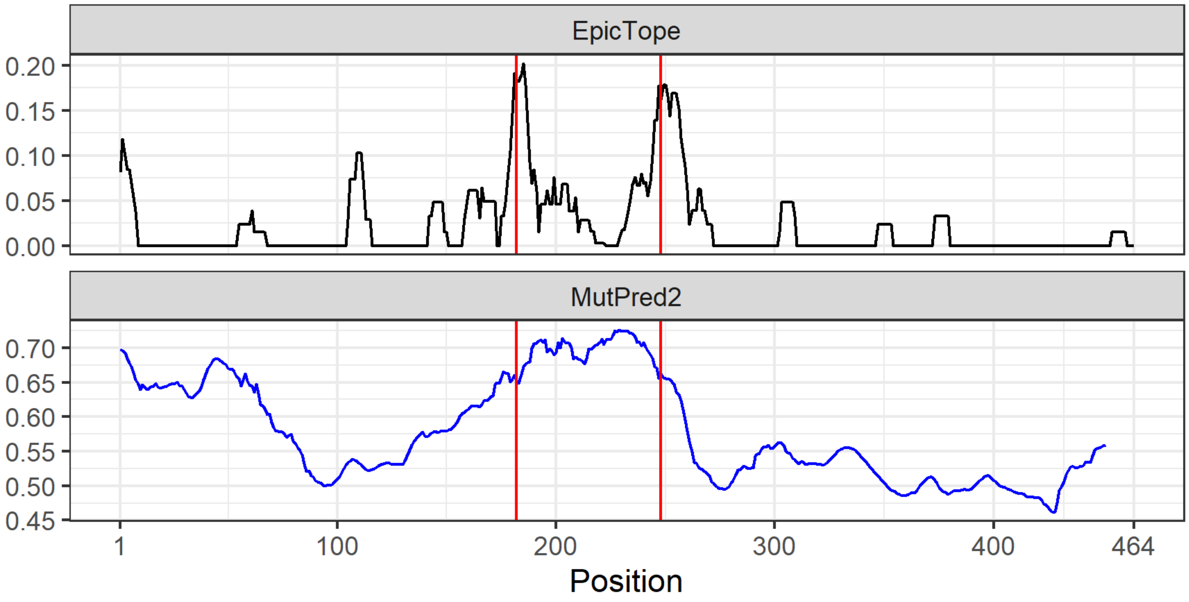

Fig. S7. Predicted Smad5 insertion sites. Shown are Mutpred2 and EpicTope prediction scores for each position in Smad5. Mutpred2 scores (blue) were generated by subtracting the Mutpred2 pathogenicity score from 1. EpicTope scores are shown in black. Red vertical lines indicate the best-suited EpicTope predicted positions, S181 and S247. Mutpred2 scores are higher for the N-terminus than either internally tagged position. Note that the y-axes scores are not comparable across methods, since MutPred provides a machine learning-based confidence score (0-1), while EpicTope has a score based on its component function as elaborated in the text. EpicTope scores across the protein sequence may vary slightly due to changes in the software comprising the pipeline (IUPred2A and reference database of DSSP). EpicTope and Mutpred2 scores are generated within a moving average window of 7 amino acids. Note that Mutpred2 does not generate scores at C-termini.

Development • Supplementary information

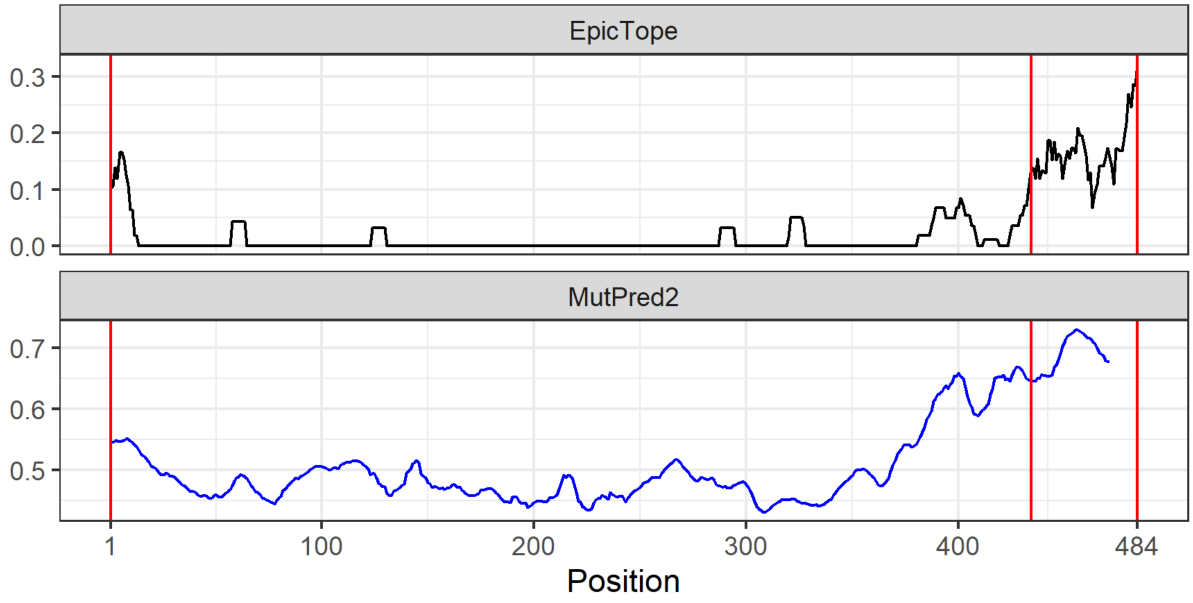

Fig. S8. Predicted Hdac1 tag insertion sites. Shown are Mutpred2 and EpicTope prediction scores for each position in Hdac1. Mutpred2 scores (blue) were generated by subtracting the Mutpred2 pathogenicity score from 1. EpicTope scores are shown in black. Red vertical lines indicate the three best EpicTope predicted tag positions, A434, as well as the N- and C-termini. EpicTope and Mutpred2 scores are generated within a moving average window of 7 amino acids. Note that Mutpred2 does not generate scores at C-termini.

Smad5 N-terminal V5 (pBB9)

```
ATGGGCAAGCCTATCCCAAACCCTCTGCTGGGCCTGGACTCCACAGGTGGTTCTGGTATGACCTCCATG
 M  G  K  P  I  P  N  P  L  L  G  L  D  S  T  G  G  S  G  M  T  S  M
                                                          1
```

Smad5 S181-V5 (pBB6)

```
CAGCAGCACAGCGGCAAGCCTATCCCAAACCCTCTGCTGGGCCTGGACTCCACAGGAGGAAGCTCC
 Q  Q  H  S  G  K  P  I  P  N  P  L  L  G  L  D  S  T  G  G  S  S
          181                                          182
```

Smad5 S247-V7 (pBB8)

```
GAGACTGGTAGCGGCAAGCCTATCCCAAACCCTCTGCTGGGCCTGGACTCCACAAGCCTGGCTCCT
 E  T  G  S  G  K  P  I  P  N  P  L  L  G  L  D  S  T  S  L  A  P
          247                                          248
```

Smad5 C-terminal V5 (pBB7)

```
TCTTCTGTCTCGGGTGGTTCTGGTGGCAAGCCTATCCCAAACCCTCTGCTGGGCCTGGACTCCACATAA
 S  S  V  S  G  G  S  G  G  K  P  I  P  N  P  L  L  G  L  D  S  T  *
          464
```

Smad5 S181-3xV5 (pKT1)

```
CAGCAGCACAGCGGCAAGCCTATCCCAAACCCTCTGCTGGGCCTGGACTCCACAGGaAAa
 Q  Q  H  S  G  K  P  I  P  N  P  L  L  G  L  D  S  T  G  K
          181

CCaATtCCcAACCCTCTcCTGGGtCTGGAtagCACcGGaAAGCCaATCCCtAAtCCcCTG
 P  I  P  N  P  L  L  G  L  D  S  T  G  K  P  I  P  N  P  L

CTcGGaCTGGACTCtACtGGAGGAAGCTCC
 L  G  L  D  S  T  G  G  S  S
                   182
```

Smad5 S247-3xV5 (pKT2)

```
GAGACTGGTAGCGGCAAGCCTATCCCAAACCCTCTGCTGGGCCTGGACTCCACAGGaAAa
 E  T  G  S  G  K  P  I  P  N  P  L  L  G  L  D  S  T  G  K
          247

CCaATtCCcAACCCTCTcCTGGGtCTGGAtagCACcGGaAAGCCaATCCCtAAtCCcCTG
 P  I  P  N  P  L  L  G  L  D  S  T  G  K  P  I  P  N  P  L

CTcGGaCTGGACTCtACtAGCCTGGCTCCT
 L  G  L  D  S  T  S  L  A  P
                   248
```

Hdac1 N-terminal V5

```
ATGGGTAAGCCTATCCCTAACCCTCTCCTCGGTCTCGATTCTACGGGTGGTTCTGGTGCTGAGTTCTCA
 M  G  K  P  I  P  N  P  L  L  G  L  D  S  T  G  G  S  G  A  E  F  S
 1
```

Hdac1 S434-V5

```
CGGAGAAACGCAGGTAAGCCTATCCCTAACCCTCTCCTCGGTCTCGATTCTACGGCCAATTACAAG
 R  R  N  A  G  K  P  I  P  N  P  L  L  G  L  D  S  T  A  N  Y  K
          434                                          435
```

Hdac1 C-terminal V5

```
TTAAAAACAGTGGGTGGTTCTGGTGGTAAGCCTATCCCTAACCCTCTCCTCGGTCTCGATTCTACGTGA
 L  K  T  V  G  G  S  G  G  K  P  I  P  N  P  L  L  G  L  D  S  T  *
          484
```

Fig. S9. Partial sequences of constructs used for rescue experiments. DNA sequences are shown above protein translation in bold. Sequences corresponding to native *smad5* and *hdac1* are highlighted in yellow, GGSG linker is highlighted in grey and V5 is highlighted in aqua. Amino acid numbers are shown in red. Lowercase letters in 3xV5 constructs indicate silent substitutions introduced to make the three tag repeats non-identical to facilitate cloning.

Development • Supplementary information

Table S1. Primers used to introduce V5 tags into *smad5* and *hdac1*. Sequences corresponding to the V5 tag are highlighted in aqua, GGSG linker - in grey, and *smad5* and *hdac1* - in yellow. Lowercase letters denote silent substitutions introduced to make sequences less repetitive.

Primer	Insertion	Sequence 5'->3'
V5Smad-F1	N-terminal V5	GGCAAGCCTATCCCAAACCCTCTGCTGGGCCTGGACTCCACAGGTGGTTCTGGTATGACCTCCATGTCTAGTCTGTT
V5Smad-R1	N-terminal V5	ACCAGAACCACCTGTGGAGTCCAGGCCCAGCAGAGGGTTTGGGATAGGCTTGCCCATAGTGCTGGGCTGCACCAGGAAG
V5Smad-F2	S181-V5	GGCAAGCCTATCCCAAACCCTCTGCTGGGCCTGGACTCCACAGGAGGAAGCTCCTTCCCCATC
V5Smad-R2	S181-V5	TGTGGAGTCCAGGCCCAGCAGAGGGTTTGGGATAGGCTTGCCGCTGTGCTGCTGGAAGGACTC
V5Smad-F3	S247-V5	GGCAAGCCTATCCCAAACCCTCTGCTGGGCCTGGACTCCACAAGCCTGGCTCCTCAGAACATG
V5Smad-R3	S247-V5	TGTGGAGTCCAGGCCCAGCAGAGGGTTTGGGATAGGCTTGCCGCTACCAGTCTCCATGGACTGA
V5Smad-F4	C-terminal V5	GGTGGTTCTGGTGGCAAGCCTATCCCAAACCCTCTGCTGGGCCTGGACTCCACATAATGATGGGCTGACCTGGGAGG
V5Smad-R4	C-terminal V5	TGTGGAGTCCAGGCCCAGCAGAGGGTTTGGGATAGGCTTGCCACCAGAACCACCCGAGACAGAAGAGATGGGGTTC
Smad5S181-3xV5-F1	S181-3xV5	ATtCCcAACCCTCTcCTGGGtCTGGAtagCACcGGaAAGCCaATCCCtAAtCCcCTGCTcGGaCTGGACTCtACtGGAGGAAGCTCCTTCCCCATC
Smad5S181-3xV5-R1	S181-3xV5	CAGaCCCAGgAGAGGGTTgGGaATtGGtTTtCCTGTGGAGTCCAGGCCCAGCAGAGGGTTTGGGATAGGCTTGCCGCTGTGCTGCTGGAAGGACTC
Smad5S247-3xV5-F1	S247-3xV5	ATtCCcAACCCTCTcCTGGGtCTGGAtagCACcGGaAAGCCaATCCCtAAtCCcCTGCTcGGaCTGGACTCtACtAGCCTGGCTCCTCAGAACATG
Smad5S247-3xV5-R1	S247-3xV5	CAGaCCCAGgAGAGGGTTgGGaATtGGtTTtCCTGTGGAGTCCAGGCCCAGCAGAGGGTTTGGGATAGGCTTGCCGCTACCAGTCTCCATGGACTG
Hdac1-F	Hdac1 ORF	CGACGCCACCATGGCGCTGAGTTCTCAAG
Hdac1-R	Hdac1 ORF	AGATCCGCCGTCACACTGTTTTTAATTCTTCTTTTGG
V5Hdac1-N-F	N-terminal V5	CCCTAACCCTCTCCTCGGTCTCGATTCTACGGGTGGTTCTGGTGCGCTGAGTTCTCAAGGAACAAAGAAGAAAGTTTGC
V5Hdac1-N-R	N-terminal V5	CCGAGGAGAGGGTTAGGGATAGGCTTACCCATGGTGGCGTCGAGTTAGATCTGCCAAAGTTGAGCG
V5Hdac1-434-F	A434-V5	CTAACCCTCTCCTCGGTCTCGATTCTACGGCCAATTACAAGAAGCCAAAACGAGTG
V5Hdac1-434-R	A434-V5	GAGGAGAGGGTTAGGGATAGGCTTACCTGCGTTTCTCCGTCCGCCCTGGC
V5Hdac1-C-F	C-terminal V5	GGTAAGCCTATCCCTAACCCTCTCCTCGGTCTCGATTCTACGTGACCGCGGATCTGGTTACCACTAAAC
V5Hdac1-C-F	C-terminal V5	GAGGAGAGGGTTAGGGATAGGCTTACCACCAGAACCACCCACTGTTTTTAATTCTTCTTTTGGCCCCTTTGTGTCCATTTTCTCTTC
pT3TS-F	pT3TS Backbone	CACCAGTCACAGAAAAGCATCTTACGGATGGCATGACAGTAAGAG
pT3TS-R	pT3TS Backbone	CGTAAGATGCTTTTCTGTGACTGGTGAGTACTC

Development • Supplementary information
